# *QuickStats:* Rate* of Unintentional Traumatic Brain Injury–Related Deaths^†^ Among Persons Aged ≤19 Years, by Age Group and Sex — National Vital Statistics System, United States, 2018–2020

**DOI:** 10.15585/mmwr.mm7111a5

**Published:** 2022-03-18

**Figure:**
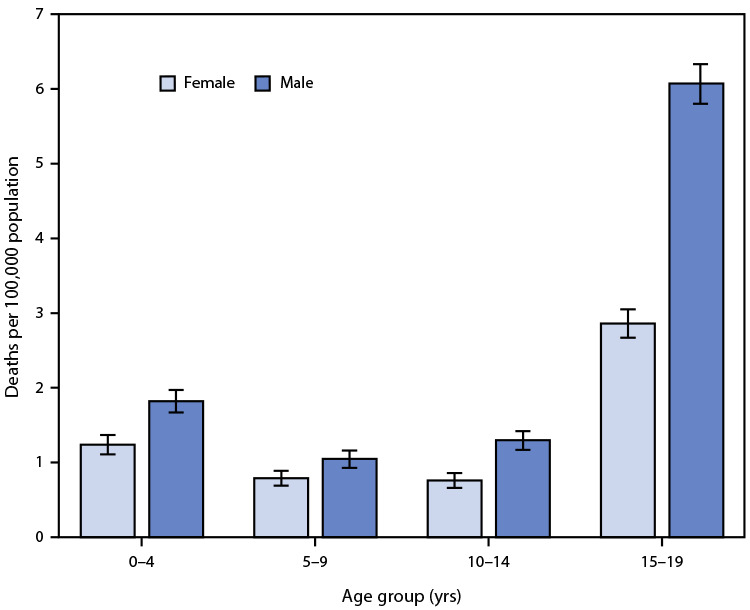
During 2018–2020, death rates for unintentional traumatic brain injury among persons aged ≤19 years were higher for males than for females in each age group. Rates were highest for males (6.1 per 100,000) and females (2.9) among persons aged 15–19 years. Rates were lowest for males and females aged 5–9 years (1.1 and 0.8, respectively) and for males and females aged 10–14 years (1.3 and 0.8, respectively).

**Source:** National Center for Health Statistics, National Vital Statistics System, Mortality Data. https://www.cdc.gov/nchs/deaths.htm

For more information on this topic, CDC recommends the following link: https://www.cdc.gov/traumaticbraininjury

